# Linking Tea Aroma Chemistry to Quality Grades via a Single MOS Gas Sensor: Classical Machine Learning vs. Deep Learning

**DOI:** 10.3390/s26123877

**Published:** 2026-06-18

**Authors:** Ahmet Turan Tasdemir, Erkan Caner Ozkat, Gozde Yalcin Ozkat, Fatih Gul

**Affiliations:** 1 Department of Mechanical Engineering, Institute of Graduate Studies, Recep Tayyip Erdogan University, Rize 53100, Türkiye; ahmetturan_tasdemir24@erdogan.edu.tr; 2 Department of Mechanical Engineering, Faculty of Engineering & Architecture, Recep Tayyip Erdogan University, Rize 53100, Türkiye; 3 Artificial Intelligence & Internet of Things Research Group, Faculty of Engineering & Architecture, Recep Tayyip Erdogan University, Rize 53100, Türkiye; 4 Department of Bioengineering, Faculty of Engineering & Architecture, Recep Tayyip Erdogan University, Rize 53100, Türkiye; gozde.yalcin@erdogan.edu.tr; 5 Department of Electrical & Electronics Engineering, Faculty of Engineering & Architecture, Recep Tayyip Erdogan University, Rize 53100, Türkiye; fatih.gul@erdogan.edu.tr

**Keywords:** black tea quality, electronic nose, MOS gas sensor, deep learning, volatile organic compounds

## Abstract

**Highlights:**

**What are the main findings?**

- A single low-cost MOS gas sensor can discriminate three commercial black tea quality grades.
- A raw-waveform deep model (MS-CNN-Attention) reached F1-macro 0.811, a 30% gain over the best classical classifier (0.624), and graded 14 of 16 products correctly by majority vote (11 of 16 for the MLP).

**What are the implications of the main findings?**

- The instrument offers a fast, non-destructive complement to sensory-panel tea grading.
- Raw-waveform modeling helps most for the medium grade (F1: 0.52 → 0.79), where summary statistics discard the release kinetics.

**Abstract:**

Black tea quality is governed by aroma chemistry: terpene alcohols (linalool, geraniol, nerolidol), methyl salicylate, and short-chain aldehydes whose abundance and release kinetics from the polyphenol-rich leaf matrix shape perceived grade. Grade information lies not only in the average headspace concentration but in the temporal shape of volatile organic compound (VOC) release under controlled heating. Conventional electronic noses obscure this signal: they rely on multi-sensor arrays, compress each response into summary statistics, and report accuracy only at the level of individual measurements. Whether a single low-cost metal–oxide–semiconductor (MOS) gas sensor can recover grade-defining aroma chemistry, and whether waveform-level modeling can exploit it, was therefore investigated. A portable electronic nose built around a Bosch BME688 sensor recorded 90 time series, each comprising four directly measured channels (temperature, humidity, pressure, gas sensor resistance) and a derived indoor-air-quality (IAQ) proxy computed from them by the on-chip BSEC library, from 16 commercial Turkish black teas across three quality grades. Two representations were compared on the same data: a feature-based pipeline reducing 25 statistical descriptors to seven principal components for six classifiers (best F1-macro = 0.624, MLP), and a raw-waveform Multi-Scale 1D-CNN with Squeeze–Excitation and temporal self-attention (MS-CNN-Attention). Under product-grouped cross-validation, the deep model reached F1-macro = 0.811 (+30%) and graded 14 of 16 products correctly by majority vote, against 11 of 16 for the MLP, with the largest gain in the medium grade (F1: 0.52 → 0.79), where summary-statistic compression destroys the release-kinetic signal. The contributions are threefold: one programmable MOS sensor operated as a thermal-desorption profiler rather than a sensor array; a direct comparison of feature-based classical learning against raw-waveform deep learning on the same small, non-normally distributed dataset; and a product-level decision-consistency metric suited to batch screening. Pairing a low-cost MOS sensor with waveform-level modeling offers a rapid, non-destructive route to aroma-chemistry-based tea quality screening.

## 1. Introduction

The sensory quality of black tea is shaped by a complex mixture of volatile and non-volatile compounds. Cultivar, terroir, and the processing stages of withering, rolling, fermentation (oxidation), and drying jointly determine the composition [1,2]. Volatile organic compounds (VOCs) are the primary carriers of tea aroma and a key quality indicator across all tea types [1,3]. Aroma dominates grade assignment. Premium teas develop rich floral and fruity notes from linalool, geraniol, and nerolidol released during controlled fermentation. Lower-grade teas yield flatter profiles with more hexanal and short-chain aldehydes [4,5]. Linalool has been identified as the key compound driving grade differentiation in oolong teas through odor activity value (OAV) analysis [4]. Perceptual interactions among key aroma compounds also give rise to synergistic or masking effects that modulate the overall sensory outcome [6]. The dynamic evolution of aroma during fermentation has been studied in ripened Pu-Erh [7] and in various black teas. These studies confirm that processing conditions strongly shape the final VOC profile. These VOC differences arise from the same enzymatic oxidation cascade that converts catechins into theaflavins and thearubigins. Those pigments govern color, astringency, and body. The headspace VOC fingerprint therefore carries information about polyphenol status and overall chemical quality [2,8]. Furthermore, the polyphenol-rich leaf matrix controls how fast individual VOCs are released when the leaf is heated. The shape of this release over time, rather than the time-averaged concentration, carries grade information: it reflects both which compounds are present and how strongly they are bound in the leaf. Broader flavor chemistry across tea types, including the interaction between volatile and non-volatile constituents, has been reviewed for specialty products such as milk tea [9]. Standardized pretreatment protocols for faithful aroma representation continue to be refined [10]. Process optimization studies on white tea further link manufacturing parameters to key flavor substances [11].

Current grading practice has two strands, and neither is fully suited to high-throughput production. Sensory panels score infusion color, aroma, taste, and mouthfeel. Their outcomes are vulnerable to taster fatigue, variation in training level, and ambient conditions [2,12]. Building robust, validated quality scoring systems remains hard even for well-characterized herbal teas [13]. Instrumental methods give accurate compound-level or spectral data but often need elaborate sample preparation, costly instrumentation, and long turnaround times. Common examples are GC-MS and GC-IMS for VOC profiling, HPLC for catechins and theaflavins, and near-infrared (NIR) spectroscopy for rapid chemical screening [2,3]. Hyperspectral imaging has shown promise for real-time tea quality prediction in cultivation settings [14]. NIR-based models can predict quality substance content in green teas [15], and shoot-trait-based models support quality evaluation of machine-picked fresh leaves [16]. Image-based approaches have been used to monitor the degree of withering [17], and three-dimensional fluorescence spectroscopy combined with UMAP enables classification of dark teas [18]. Despite this range of methods, rapid on-line aroma-based screening of finished tea products is still missing.

Electronic nose (e-nose) technology has emerged as a promising route to close this gap. Early multi-sensor MOS arrays already discriminated tea grades from headspace VOC patterns, first with neural networks [12] and LDA/PCA [19], and later with chemometric classifiers such as PCA, LDA, and SVM for in situ black-tea grading [20] and with extreme learning machines for tea-gas identification [21]. To push accuracy higher, several groups fused the e-nose with a second modality, such as Vis–NIR spectroscopy [22], hyperspectral imaging through global–local feature-fusion networks [23], molecularly imprinted electronic tongues [24], or combined computer-vision, e-nose, and e-tongue platforms for storage-life prediction [25]. Even low-cost hardware can give competitive discrimination when paired with suitable machine learning (ML) pipelines such as PLSDA, LDA, and PCA [26], while portable aroma sensors [27] and adaptive gas-feature networks for origin traceability [28] point to a clear trend toward compact, field-deployable instruments. Deep learning has since been adopted widely in the tea domain, including cross-time–frequency networks for agricultural-product recognition [29], two-dimensional correlation spectroscopy for high-precision black-tea classification [30], multi-view multi-task networks for joint classification and flavor-factor estimation [31], transfer learning on NIRS data [32], convolutional networks for rapid green tea quality prediction [33], ML-based taste-profile classification [34], and lightweight networks for leaf-appearance inspection [35]. Methods from neighboring fields transfer directly to sensor signals as well, such as augmentation strategies from hyperspectral remote sensing [36] and deep architectures for time series classification [37].

Despite these advances, four main limitations persist: (i) most e-nose platforms use arrays of 6–16 sensors, which adds cost and system complexity, leaving open whether a single programmable MOS sensor with multiple heater channels can give useful grade discrimination; (ii) evaluation has mostly looked at instance-level accuracy and has overlooked whether the instrument gives consistent verdicts when the same tea product is retested across multiple sessions, which is a basic requirement for on-line quality control; (iii) feature-based approaches reduce sensor waveforms to summary statistics such as means and standard deviations, discarding the temporal shape of the VOC-release curves, and because gas-resistance readings often have heavy tails and pronounced skewness, losing this kinetic information can be decisive for classification performance; and (iv) no direct side-by-side comparison of classical ML and deep learning has been reported for single-sensor tea grading on a small, non-normally distributed dataset.

These limitations are addressed here with a portable platform built around a single Bosch BME688 sensor (Bosch Sensortec GmbH, Reutlingen, Germany). The framing is explicit: the aim is to recover grade-defining aroma chemistry from a low-cost sensor trace. The principal contributions are: (i) a portable single-sensor platform that performs controlled thermal VOC desorption from dry tea, designed to record chemically interpretable release-kinetic traces rather than time-averaged headspace concentrations; (ii) a six-model classical ML benchmark that quantifies how much aroma-chemistry information is preserved when sensor waveforms are compressed into PCA-reduced statistical features; (iii) a Multi-Scale 1D-CNN with Squeeze–Excitation and temporal self-attention (MS-CNN-Attention) that operates directly on the raw VOC-release waveforms and, by learning from the full trace, retains the release-kinetic and signal-shape information that per-channel summary statistics discard; and (iv) a product-level decision consistency metric that quantifies prediction stability across repeated measurements of the same tea product, directly relevant to on-line aroma-based quality screening in tea processing facilities.

The novelty of this work therefore does not lie in proposing a new sensor or a new generic classifier. It lies in the specific combination, not previously reported for tea grading, of (i) a single programmable MOS sensor used as a thermal-desorption profiler instead of a multi-sensor array, (ii) a head-to-head comparison of feature-based classical ML against raw-waveform deep learning on the same small, non-normally distributed dataset, and (iii) a product-level decision-consistency analysis that evaluates the instrument as a screening tool rather than only at the level of individual measurements.

The remainder of the manuscript is organized as follows. Section 2 describes the tea samples, the single-sensor platform, and the measurement protocol. Section 3 details the two analytical pipelines: a feature-based classical ML pipeline and a raw-waveform deep learning model. Section 4 reports the classification and product-level consistency results for both paradigms. Section 5 interprets these findings in terms of VOC-release kinetics and aroma chemistry, examines sensor drift and calibration, and contrasts the design choices with representative prior tea e-nose studies. Section 6 draws the main conclusions.

## 2. Materials

### 2.1. Tea Samples

Sixteen commercially sourced dry black tea products (A1–A16) were selected from the Turkish market, spanning three quality grades: high (Class 1, 5 products), medium (Class 2, 6 products), and low (Class 3, 5 products). The selection includes loose-leaf, tea-bag, and cup-bag formats, as well as natural and bergamot-flavored varieties. Quality class labels were assigned using three chemical indicators that are widely used in the tea science literature [1,2,4]: (i) total polyphenol content covering catechin, theaflavin, and thearubigin fractions; (ii) ORAC (Oxygen Radical Absorbance Capacity) antioxidant capacity; and (iii) caffeine concentration. These values came from manufacturer declarations, package labels, and published analytical data. Independent chemical analyses were not run on the specific samples used here. Table 1 summarizes the average chemical and sensory profiles for each grade, and Table 2 lists all products.

### 2.2. Sensor Platform and VOC Sampling

**Figure 1:**
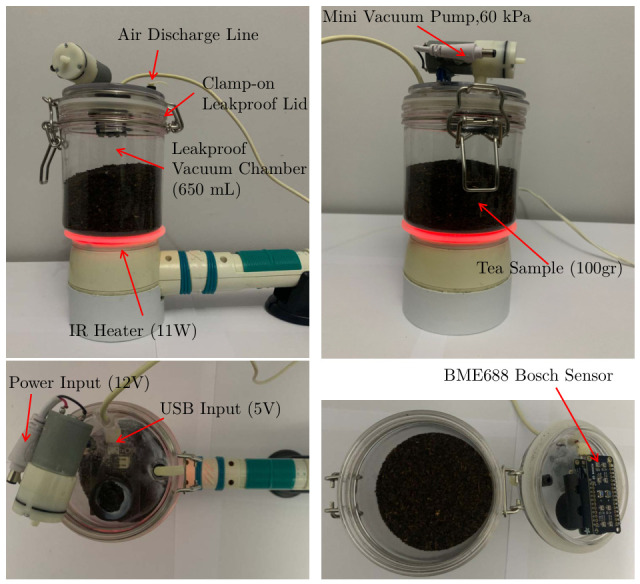
Portable e-nose platform. The sealed chamber concentrates headspace VOCs at the BME688 sensor under controlled infrared heating and vacuum conditions.

The instrument (Figure 1) integrates five components: a Bosch BME688 MOS gas sensor (Bosch Sensortec GmbH, Reutlingen, Germany) with eight programmable heater set-points; an Adafruit HUZZAH32 ESP32 microcontroller (Adafruit Industries, New York, NY, USA) for I^2^C data acquisition and microSD logging; a sealed 650 mL glass chamber; an 11 W infrared heater; and a 12 V vacuum pump. The BME688 directly measures four physical quantities—temperature, relative humidity, pressure, and gas sensor resistance—from which the on-chip BSEC library additionally computes an indoor-air-quality (IAQ) index used as a fifth, derived channel [38,39]. The heater raises the tea temperature to 30–50 °C, promoting thermal desorption of VOCs from the leaf surface into the headspace, while the vacuum pump concentrates the resulting vapors at the sensor surface [22].

The Bosch BME688 MOS gas sensor was selected rather than dedicated digital gas sensors such as the Sensirion SGP41 (Sensirion AG, Stäfa, Switzerland) or the ScioSense ENS16x family (ScioSense B.V., Eindhoven, The Netherlands) for three reasons. First, it integrates gas, temperature, humidity, and pressure transducers in a single package, which allows the gas response to be compensated for ambient temperature and humidity without additional sensors. Second, and most important for this study, its eight independently programmable heater set-points give direct access to the raw gas sensor resistance under a user-defined thermal profile; this is what makes waveform-level thermal-desorption profiling possible from one sensing element, effectively turning a single sensor into a virtual multi-sensor through temperature modulation. Sensors that expose only a preprocessed VOC or air-quality index (as the SGP41 and ENS16x families primarily do) would not provide the raw release-kinetic trace on which the analysis here depends. Third, the BME688 is low-cost, compact, and widely available, which is consistent with the goal of a portable, inexpensive screening instrument.

### 2.3. Measurement Protocol

**Figure 2:**
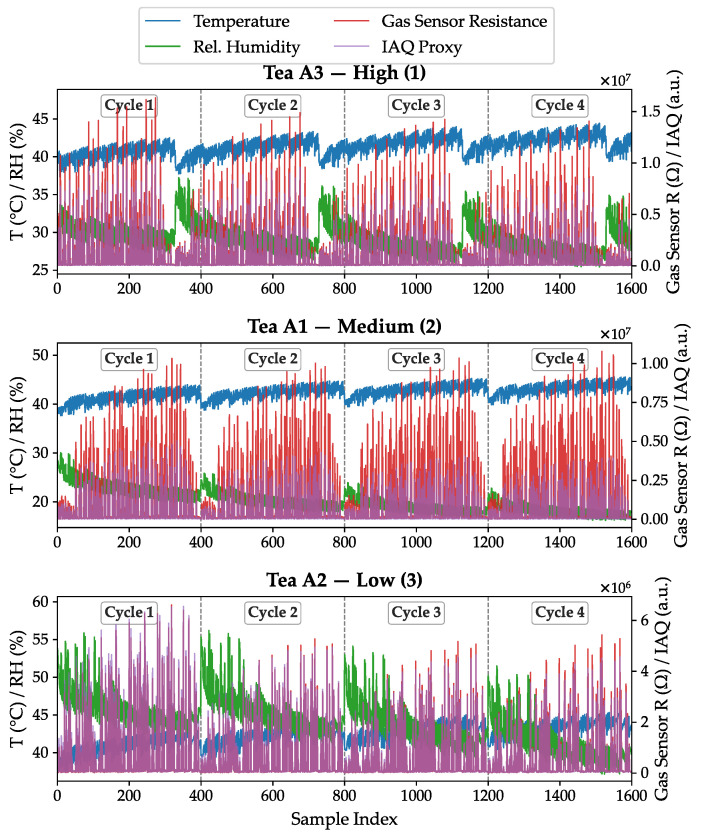
Raw sensor time series over the first four heater-measurement cycles (the leading ∼1600 samples) of one representative run per quality grade; each heater super-cycle spans ∼400 samples (∼1 min) and cycle boundaries are marked by dashed lines. Temperature and relative humidity are plotted on the left axis. Gas sensor resistance and the IAQ proxy (right axis) reflect headspace VOC concentration and display the most pronounced grade-dependent differences.

Each measurement run began with a 30 min baseline acquisition in the empty sealed chamber. A 30 min exposure phase with about 100 g of tea then followed, with the heater and vacuum pump active. The sensor sampled at around 1.38 Hz across both phases, yielding 3600–5800 data points per channel for each complete run. A 30 min clean-air purge was applied between successive runs to prevent cross-contamination [39]. In total, 90 runs were collected (30 high-grade, 29 medium-grade, 31 low-grade). Figure 2 shows representative waveforms. The gas sensor resistance and IAQ-proxy channels are most directly coupled to headspace VOC concentration. They also show the strongest inter-class separation.

**Figure 3:**
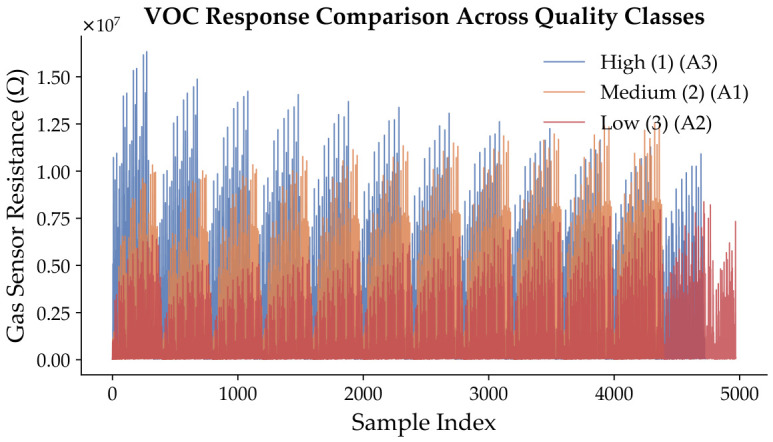
Overlay of gas sensor resistance time series from one representative sample per quality grade. The high-quality tea produces a stronger and more sustained sensor response than the medium and low grades, reflecting differences in VOC concentration, composition, and release kinetics.

To isolate the most aroma-relevant channel, Figure 3 overlays the gas sensor resistance traces from one representative sample of each grade on a common time axis. The high-quality tea (A3, Class 1) shows a sharp initial rise and then a sustained plateau at elevated resistance. This fits the slow, prolonged release of terpene alcohols such as linalool and geraniol from a polyphenol-rich leaf matrix. The medium-grade sample (A1, Class 2) has a lower peak amplitude and a faster decay rate, in line with its intermediate VOC load. The low-quality tea (A2, Class 3) gives the weakest and most rapidly fading response, consistent with a sparser headspace dominated by lighter, faster-desorbing aldehydes. These distinct temporal profiles confirm that the gas sensor resistance channel alone carries substantial grade-discriminative information. They are the main reason full-waveform classification is used rather than scalar summary statistics.

## 3. Methods

Two independent analytical pipelines were applied to the same dataset: (A) a feature-based classical ML pipeline, and (B) a raw-waveform deep learning pipeline. In both cases, the complete five-channel record of each run (baseline plus exposure) was used.

### 3.1. Pipeline A: Feature-Based Classical ML

**Figure 4:**
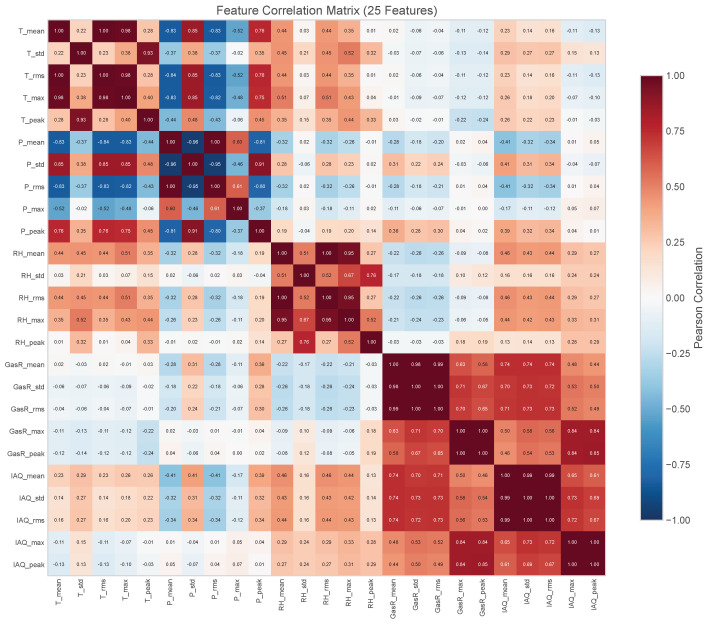
Pearson correlation coefficients among the 25 extracted features. Substantial within-sensor redundancy (*r* > 0.95) justifies dimensionality reduction via PCA prior to classification.

**Figure 5:**
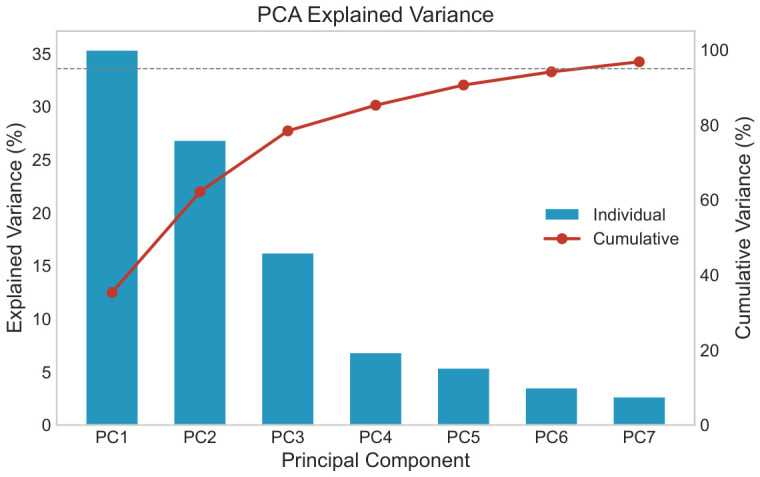
Cumulative explained variance of the PCA components. Seven components collectively retain 96.8% of total variance (dashed line: 95% threshold).

Five time-domain statistical descriptors were computed for each of the five sensor channels: mean, standard deviation, root mean square (RMS), maximum, and peak deviation, where peak deviation is defined as the signal range, max–min, of the channel over the run. This produced a 25-dimensional feature vector per run. Z-score normalization and principal component analysis (PCA) were then applied, keeping seven components to cover 95% of the explained variance. Figure 4 shows the feature correlation matrix. The strong within-group redundancy (*r* > 0.95 among gas sensor resistance features) confirms that the PCA reduction step was needed. Figure 5 shows the cumulative explained-variance profile.

Six classifiers were benchmarked: SVM (one-vs.-one) [40], k-NN, Random Forest [41], XGBoost [42], LightGBM [43], and MLP. Benchmarking practices established for multi-class sensor-signal classification problems were followed [44]. Each model was optimized with a 5-fold inner grid search on F1-macro and evaluated under 10-fold stratified outer cross-validation [45,46]. Table 3 lists the hyperparameter search grids.

### 3.2. Pipeline B: Raw-Waveform Deep Learning

The feature-based pipeline gave the wrong dominant class label for 5 of 16 products (Section 4.2). This indicates that classification-relevant information is lost when sensor waveforms are compressed into five per-channel summary statistics. Similar limitations have been reported in other sensor domains, where working directly on raw signal patterns gives better discrimination than hand-crafted descriptors [47]. In this dataset, gas sensor resistance traces are markedly right-skewed and heavy-tailed. This pattern reflects the burst-then-plateau kinetics typical of VOC desorption from the tea leaf matrix. Mean and standard deviation alone cannot separate teas whose aroma-release profiles differ even when their average headspace concentrations are similar. Accordingly, a deep learning model that operates on the full release waveform without prior feature extraction was developed.

#### 3.2.1. Input Representation and Data Augmentation

Each run was resampled to a uniform length of (5 × 4400) data points by linear interpolation. Per-channel z-scores were computed from training-fold statistics and applied consistently to the validation folds. Several training-time augmentations were used in random combinations of two to three operations per sample. The augmentations were Gaussian jitter (*σ* = 0.03), per-channel random scaling (*σ* = 0.1), magnitude warping (4 control knots, *σ* = 0.2) [48], temporal permutation, and 90%-crop-resize. Here temporal permutation does not shift the waveform globally; instead, it divides each run into a small number of contiguous equal-length segments and randomly permutes the order of those segments (local block shuffling), so that intra-segment release kinetics are preserved while their global ordering is perturbed. This operation was applied to a given training sample with probability *p* = 0.3. Mixup regularization (*α* = 0.3, *p* = 0.7) [49] added further implicit regularization during training.

#### 3.2.2. Architecture: MS-CNN-Attention

**Figure 6:**
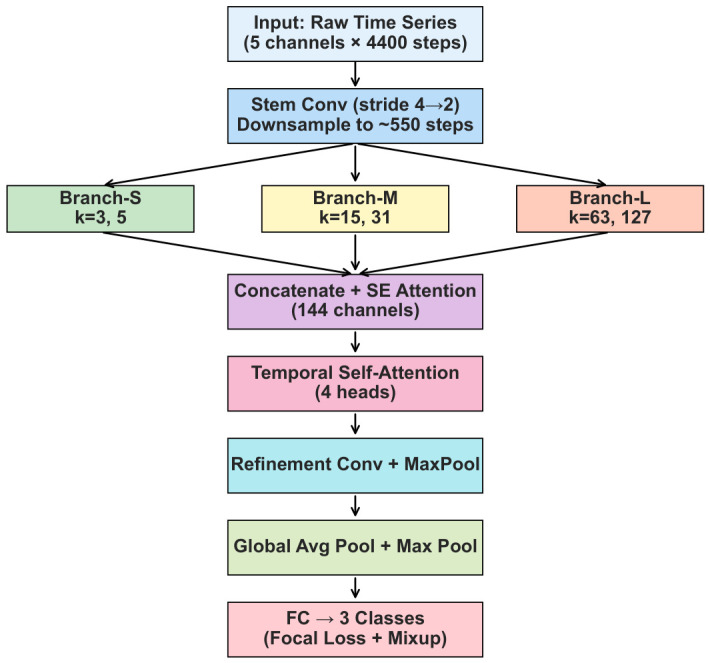
Schematic of the MS-CNN-Attention architecture. A stem module downsamples the input; three parallel branches extract short-, mid-, and long-range temporal features with SE channel attention; multi-head self-attention aggregates the branch outputs; dual global pooling feeds a three-class classification head.

The architecture (Figure 6) processes the input waveform in four sequential stages. The first is a stem of two strided convolutions (*s* = 4, 2) that reduces the 4400-step input to about 550 steps and expands the 5 input channels to 64 feature maps (BatchNorm + GELU activation). The second stage is three parallel multi-scale branches with kernel sizes of (3, 5), (15, 31), and (63, 127). These are intended to capture fast transient VOC spikes, gradual terpene-alcohol build-up curves, and slow sesquiterpene emission trends. Each branch is two Conv1D–BatchNorm–GELU–Dropout–Squeeze–Excitation (SE) [50] blocks followed by max-pooling. The SE gates learn to weight sensor-derived feature channels by their aroma-discriminative relevance for each sample. The third stage is a 4-head self-attention layer [51] applied across the temporal axis. It assigns higher weights to time steps with greater class-discriminative value. VOC release peaks in the mid-window phase as the leaf temperature rises, so the attention mechanism learns to up-weight this interval on its own. Manual window selection is not needed. The fourth stage is two additional convolutional blocks, global average and max-pooling (giving a 128-dimensional representation), and three fully connected layers (GELU activation, dropout) that output 3-class logits.

#### 3.2.3. Training Configuration

Focal loss [52] (*γ* = 2, class-frequency-weighted *α*) was used to push gradients toward hard boundary samples and to soften the effect of class imbalance. Pairing a domain-informed loss function with data-driven parameter tuning follows the analytical-to-AI modeling strategy described by Altunkaya et al. [53]. Optimization used AdamW [54] at an initial learning rate of 10^−3^ and weight decay of 10^−3^ on a cosine annealing schedule, with a batch size of 16 and gradient clipping at a maximum norm of 1.0. Training was stopped with early stopping (patience 30 epochs, maximum 150 epochs), monitoring validation F1-macro.

StratifiedGroupKFold cross-validation (*k* = 10, grouped by product identifier) was applied. This ensures that all measurement runs of a given tea product fall into either the training or the test partition within each fold. It prevents data leakage from repeated measurements of the same product.

#### 3.2.4. Comparability of the Validation Protocols

The two pipelines use different cross-validation protocols, and this is stated explicitly to avoid any ambiguity. The classical models were evaluated with standard 10-fold stratified cross-validation, which balances class proportions across folds but does not group runs by product. The deep learning model was evaluated with the stricter StratifiedGroupKFold protocol, in which all runs of a given product are confined to a single fold. The grouped protocol is the more conservative of the two: it removes any possibility that near-identical repeated runs of the same product appear in both the training and the test partition. The comparison is therefore not biased in favor of the deep learning model. If anything, the classical baselines were evaluated under the more permissive (non-grouped) protocol, so the superior performance of the deep learning model cannot be an artifact of an easier validation split. The per-product consistency reported in Section 4.2 and Section 4.3 aggregates each fold’s held-out predictions per product under the respective protocol of each pipeline.

To verify this empirically rather than by argument alone, all six classical models were re-evaluated under the identical StratifiedGroupKFold protocol used for the deep model, keeping the same 25-feature, PCA-reduced pipeline. Under the product-grouped protocol, the classical F1-macro falls to 0.28–0.46 (best: XGBoost, 0.46), compared with the 0.51–0.62 obtained under non-grouped stratified CV (Table 4). This drop is expected: when repeated runs of the same product are confined to a single fold, feature-based models can no longer exploit product-identity cues shared between near-duplicate runs split across training and test. The deep model, evaluated under this same grouped protocol throughout, attains an F1-macro of 0.811. The two pipelines are therefore directly comparable under the stricter protocol, and the deep model’s advantage is conservative rather than inflated: under a fair, identical validation split the gap between the raw-waveform model and the best feature-based baseline widens substantially.

## 4. Results

### 4.1. Classical ML Performance

Table 4 summarizes the 10-fold cross-validation results for all six feature-based classifiers. F1-macro scores ranged from 0.505 (LightGBM) to 0.624 (MLP). Two findings stand out. The medium-grade class was the hardest for every classifier (per-class F1: 0.43–0.58). Its polyphenol content and aroma chemistry sit between the other two grades. These products share VOC traits with both high- and low-grade neighbors, so the 25-dimensional statistical feature space has limited discriminating power in this mid-range region. Among the gradient-boosted ensembles, LightGBM gave the lowest F1-macro (0.505): the small training partition (about 81 samples per fold) does not support the deep decision-tree structures these methods favor, and only grid-searched regularization kept XGBoost (0.604) competitive with the simpler classifiers.

**Figure 7:**
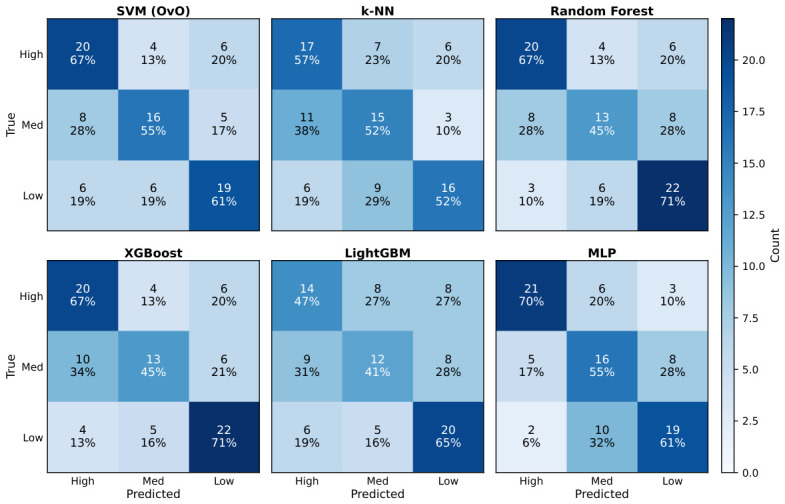
Confusion matrices for all six classical models (10-fold cross-validation, *n* = 90). Cell values indicate counts and row-normalized percentages. The medium-grade class is the most frequently misclassified across all models.

The confusion matrices in Figure 7 show a consistent error pattern across all six classifiers. The high- and low-quality classes sit on the diagonal with moderate correct classification counts. The medium-grade samples spread across all three predicted labels. Medium-grade teas are misclassified as high-grade somewhat more often than as low-grade (aggregated over the six models, 51 vs. 38 of the medium-grade misclassifications). This asymmetry indicates that the statistical feature vectors of mid-grade aroma profiles partially overlap with those of premium teas. This is not surprising. Medium-grade loose-leaf products (A1, A4, A5) share similar polyphenol ranges with the lower boundary of the high-quality group. MLP gives the strongest overall diagonal (70%, 55%, 61%); LightGBM shows the weakest separation on the high and medium grades (47%, 41%, 65%).

### 4.2. Product-Level Consistency: Classical ML

Instance-level accuracy alone does not reveal whether the system gives stable verdicts when the same tea product is retested across multiple sessions on different days. That stability is a basic practical requirement for production-line deployment. Accordingly, a product’s consistency rate is defined as the fraction of its repeated runs assigned to the most frequently predicted (dominant) class. Table 5 shows the per-product results for the best-performing classical model, MLP.

Five of sixteen products got the wrong dominant label (highlighted in bold in Table 5). The failures fall into two kinds. Three medium-grade teas (A4, A5, A12) were systematically pushed into Class 1. Their loose-leaf format and moderate polyphenol concentrations produce statistical feature vectors that sit close to the high-grade cluster, and the PCA projection cannot resolve this overlap. The other two failures are different. A15 (medium-grade, bag format) was unanimously misclassified as Class 3, and A13 (low-grade) showed near-random scatter (40% consistency). These results expose a ceiling for the feature-based approach. When VOC concentration averages overlap across quality grades, five per-channel summary statistics do not preserve enough distributional information to separate teas whose release kinetics are the main source of class-discriminative variation.

### 4.3. Deep Learning Results

Table 6 shows the per-product consistency of MS-CNN-Attention under StratifiedGroupKFold 10-fold cross-validation. The deep learning model grades 14 of the 16 products correctly by majority vote and recovers three of the five products that the MLP baseline assigns to the wrong grade: A10 (high-grade loose-leaf) and A4 and A15 (medium-grade loose-leaf and bag teas). All three are unflavored products, which suggests that the raw waveform captures release-shape cues that per-channel summary-statistic features do not represent.

Two classification errors persist. Sample A12 is a bergamot-flavored bag product. It contains bergamot oil whose dominant monoterpenes (limonene, linalyl acetate) produce a headspace fingerprint that resembles those of high-grade floral teas rather than the medium-grade bag base it is built on. Sample A13 shows near-random scatter (40% consistency) across all three classes. Its processing-derived aroma compounds fall outside the patterns the model learned from the remaining 15 products. Both persistent errors share a common cause. They involve exogenous aromatic or processing-derived compounds that override the natural VOC fingerprint expected for a given quality grade.

**Figure 8:**
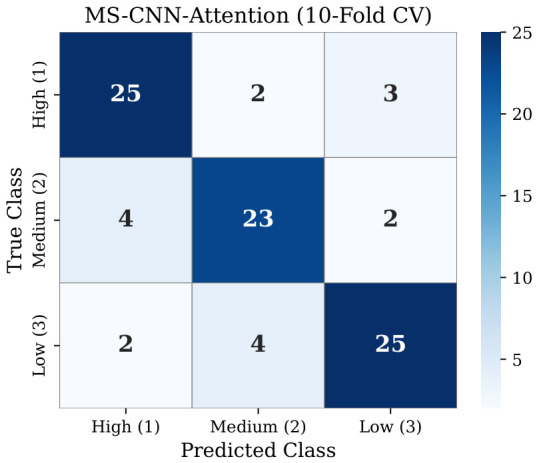
Confusion matrix for MS-CNN-Attention (aggregated 10-fold cross-validation, *n* = 90). Off-diagonal counts are reduced in every cell relative to the best classical model (MLP).

**Figure 9:**
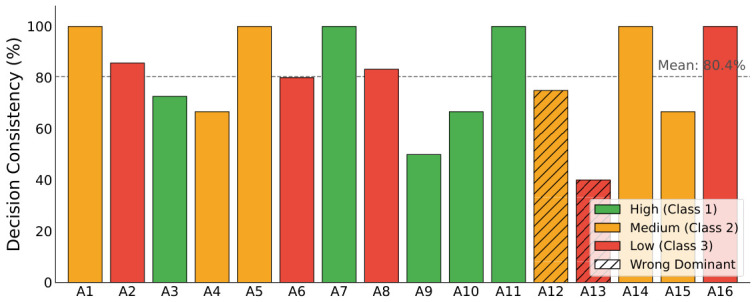
Per-product consistency for MS-CNN-Attention. Color encodes the true quality class; hatching identifies products for which the dominant predicted class is incorrect.

The confusion matrix (Figure 8) shows that the deep learning model reduces off-diagonal counts in every cell relative to MLP. The medium-grade class improves from 16/29 to 23/29 correctly classified instances. The per-product consistency chart (Figure 9) confirms that the gains are not coming from a small number of favorable folds. Products A1, A5, A7, A14, and A16 each reach 100% consistency.

### 4.4. Overall Comparison

Table 7 consolidates the results for all seven models. The deep learning model improves F1-macro by 30% and lifts the number of correctly dominant-assigned products from 11/16 to 14/16 relative to MLP. The gain is not uniform across classes. The high grade, already the easiest for the feature-based models, improves modestly (0.73 → 0.82). The medium grade—the lowest-scoring class for every classical model—rises from 0.52 to 0.79 (+51%), and the low grade improves comparably (0.56 → 0.82). These larger jumps in the two weakest classes indicate that the raw waveform keeps grade-relevant kinetic information that is most useful exactly where summary statistics fail, in the overlapping mid-to-low region of the aroma-chemistry spectrum. The higher fold-to-fold variance of the deep learning model (±0.198 vs. ±0.133 for MLP) is a built-in trade-off of training a higher-capacity model on 90 samples. Even so, its aggregated classification metrics and product-level consistency are clearly better across all evaluation criteria.

## 5. Discussion

### 5.1. VOC-Release Kinetics and the Advantage of Raw-Waveform Modeling

When dry tea is heated to 30–50 °C inside a sealed chamber, VOCs desorb from the leaf matrix in a burst-then-decay pattern. The shape of that pattern depends on each compound’s vapor pressure, the available leaf-surface area, and the strength of the polyphenol–VOC binding matrix [1,4]. Premium teas carry high linalool and geraniol loads bound within a polyphenol-rich scaffold. They release these terpene alcohols more slowly and at higher peak concentrations than lower-grade products, whose headspace is dominated by lighter, faster-desorbing short-chain aldehydes. The gas sensor resistance waveform recorded by the BME688 folds these desorption kinetics into a single temporal trace. The grade-relevant signal is in the morphology of that trace. The mean or maximum amplitude alone is not enough.

Compressing this waveform to five per-channel summary statistics collapses the shape into scalar descriptors. For roughly symmetric, unimodal signals that representation can be enough. The gas sensor resistance traces in this dataset are different. They are markedly right-skewed, since the initial desorption burst produces a pronounced long tail. Consequently, mean and standard deviation cannot separate teas whose average VOC headspace concentrations are similar but whose temporal release profiles diverge. The convolutional neural network operates on the full 4400-point waveform. It keeps this kinetic information and learns shape-sensitive filters end-to-end.

The multi-scale branch architecture mirrors the multi-timescale nature of VOC release from the leaf matrix. Small kernels (3–5 samples) resolve fast transient spikes from highly volatile aldehydes. Medium kernels (15–31) track the gradual accumulation of terpene alcohols. Large kernels (63–127) encode the slow emission tail dominated by heavier sesquiterpenes [37]. Temporal self-attention further sharpens grade discrimination. It assigns higher weights to the mid-window interval where VOC release peaks and suppresses contributions from the uninformative early warm-up and late steady-state plateaus.

### 5.2. Aroma Chemistry and Residual Classification Errors

High-grade bud teas (ORAC 1050–1250 μmol TE/100 mL) carry high catechin and theaflavin concentrations. They give complex, high-intensity headspace fingerprints dominated by floral terpene alcohols [2,4]. The sensor platform consistently separates these from low-grade CTC and bag-format teas, whose headspace is both weaker and less chemically differentiated. The medium-grade class sits in a transitional zone in polyphenol content and VOC diversity. It is the hardest class to resolve. The deep learning model still narrows this gap substantially (F1: 0.52 → 0.79) by exploiting release-shape differences that summary-statistic representations cannot see.

The two persistent misclassification cases involve products carrying aromatic compounds unrelated to the native leaf chemistry. Sample A12 is a bergamot-flavored bag product. It contains bergamot oil whose dominant monoterpenes (limonene and linalyl acetate) produce a headspace fingerprint similar to those of high-grade floral teas rather than the unflavored medium-grade bag substrate it is built on. Sample A13 also seems to carry processing-derived aroma compounds that shift its sensor fingerprint away from the unflavored members of its assigned quality class. Fixing such cases would need either a preprocessing stage that normalizes for exogenous flavoring agents or an auxiliary classifier that first detects non-tea aromatic additives before the grade-assignment model is applied.

### 5.3. Bridging Instrumental and Sensory Evaluation

Trained sensory panels assess aroma, color, astringency, and body. These attributes are rooted in the same polyphenol and VOC chemistry that the BME688 captures. A single-sensor electronic nose cannot replace a trained sensory panel. It can, however, work as a fast screening instrument. It can flag batches whose headspace fingerprint deviates from the expected grade profile and so reduce the number of samples that need full panel-based evaluation. The product-level consistency metric introduced here speaks directly to this screening role. The system grades 14 of 16 products correctly by majority vote. In a production-line context, each batch is usually evaluated across multiple measurement sessions. A system that occasionally gets an individual run wrong but consistently assigns the correct grade at the product level is still useful and deployable.

### 5.4. Sensor Drift and Calibration

Like all metal–oxide gas sensors, the BME688 is subject to drift. Drift is a slow change in the baseline gas sensor resistance over days to months, caused by aging of the SnO_2_-based sensing layer, gradual poisoning by ambient contaminants, and residual humidity effects. Its practical consequence for the algorithms used here is distributional: drift shifts the absolute resistance level and therefore the statistical features (mean, RMS, maximum) on which the classical pipeline relies, so a model trained on data recorded at one time can degrade when applied to data recorded weeks later, even for the same tea. Two aspects of the design adopted here limit this effect. First, every exposure run is referenced to a freshly recorded 30 min empty-chamber baseline, so the discriminative information lies in the relative change and in the temporal shape of the response rather than in its absolute level; relative, baseline-referenced features are inherently less sensitive to slow absolute drift. Second, the deep model is trained on the full waveform morphology, which is more robust to a constant baseline offset than scalar amplitude features.

Calibration in this context consists of re-establishing the clean-air baseline of the sensor. In each run, this is done automatically: the on-chip BSEC library continuously tracks the clean-air reference, and the 30 min empty-chamber acquisition provides a per-run zero against which the subsequent exposure is measured. A full recalibration simply repeats the clean-reference-air measurement to reset the baseline; it requires only clean air and a few minutes, with no specialized reagents, but it must be repeated periodically for long-term field use. Learning-based drift handling is also attractive: domain adaptation and drift compensation schemes, combined with the baseline-referenced features already used here, can absorb part of the drift and so reduce how often manual recalibration is needed. A systematic drift compensation study over extended deployment windows is left for future work.

### 5.5. Comparison with Prior Tea e-Nose Studies

Table 8 positions this work against representative tea e-nose studies. Three points stand out. First, the great majority of prior systems use multi-sensor arrays or fuse the e-nose with a second modality such as NIR or hyperspectral imaging [22,23,26]; here, a single programmable MOS sensor, operated as a thermal-desorption profiler, provides the discriminative signal. Second, most studies classify hand-crafted features with classical chemometrics (PCA, LDA, SVM, ELM) [12,19,20,21,26], whereas recent deep models are typically reported on their own rather than benchmarked against a feature-based baseline on the same data [29,30]. The two paradigms are instead compared directly on one small, non-normally distributed dataset. Third, evaluation in prior work is almost exclusively at the level of individual measurements; to the authors’ knowledge, no earlier tea e-nose study reports a product-level decision-consistency analysis, which is the metric most relevant to deployment as a batch-screening instrument.

### 5.6. Limitations

1. **Dataset size.** A dataset of 90 runs from 16 products is small for deep learning applications. The elevated fold-to-fold F1-macro variance reflects this. Expanding the dataset to include more products and more replicate measurements would improve model stability and generalization.
2. **Flavored tea products.** Bergamot and other exogenous aromatic additives confound the natural VOC fingerprint. A dedicated detection or normalization procedure will be needed to handle this class of samples.
3. **Single-sensor configuration.** A multi-sensor array with complementary chemical selectivity could improve class separability. The trade-off is the current platform’s simplicity and cost-effectiveness.
4. **Absence of direct chemical validation.** The quality class labels were derived from published literature and manufacturer data rather than from HPLC or GC-MS analyses of the specific samples used. Pairing future sensor runs with GC-MS headspace profiling would strengthen the mechanistic link between the sensor response and individual VOC species.
5. **Sensor drift.** Long-term sensor stability over extended deployment windows was not evaluated. As discussed in Section 5.4, the baseline-referenced acquisition limits the impact of slow drift, but periodic recalibration and, ideally, learning-based drift compensation will be needed for sustained field deployment.

## 6. Conclusions

Six classical ML classifiers and a purpose-built deep learning architecture (MS-CNN-Attention) were compared for quality-grade classification of black tea from the headspace VOC fingerprint captured by a single BME688 MOS gas sensor. The classical models worked on 25 statistical features reduced to seven principal components by PCA. They reached F1-macro values in the range 0.51–0.62. The deep learning model was trained end-to-end on raw five-channel VOC-release waveforms with focal loss, mixup augmentation, and product-grouped cross-validation. It reached an F1-macro of 0.81 (+30% relative to MLP) and correctly assigned 14 of 16 products by majority vote, against 11 of 16 for MLP. The improvement is largest for the medium-grade class (F1: 0.52 → 0.79). That is exactly where aroma chemistry overlaps most with neighboring grades and where summary-statistic compression discards the most release-kinetics information.

The platform offers a fast, non-destructive, low-cost complement to sensory panel evaluation. It is well suited to screening incoming tea lots at processing facilities. Future work will pair sensor recordings with GC-MS headspace analysis to establish mechanistic links between individual VOC species and the features learned by the CNN. The dataset will also be expanded to cover teas from diverse origins and harvest years, and lightweight model architectures suitable for real-time edge deployment will be explored.

## Figures and Tables

**Table 1:** Average chemical and sensory profiles of the three tea quality classes. Values are indicative ranges compiled from manufacturer data and the literature.

Class	Label Level	Polyphenol	ORAC (μmol TE/100 mL)	Caffeine (mg/100 mL)	Typical Characteristics
1	High	High	1050–1250	30–45	Premium buds, rich aroma, organic
2	Medium	Moderate	800–1050	25–40	Standard leaves, balanced profile
3	Low	Low	500–800	20–35	CTC/bag form, flavored, weak aroma

**Table 2:** Tea samples grouped by quality class. ORAC ranges are indicative values from manufacturer data and the literature.

Code	Form	Class	ORAC (μmol TE/100 mL)	Key Feature
A3	Loose	1	1050–1250	Premium buds, high EGCg
A7	Loose	1	∼1250	High theaflavin
A9	Bag	1	1000–1200	Premium grade, whole leaf
A10	Loose	1	1050–1250	Premium loose leaf
A11	Bag	1	1000–1200	High-grade, rich aroma
A1	Loose	2	800–1000	Fine-sieved leaves
A4	Loose	2	800–1050	Young shoots
A5	Loose	2	850–1050	Balanced profile
A12	Bag	2	800–1050	Bergamot-flavored
A14	Loose	2	800–1000	Organic, additive-free
A15	Bag	2	800–1000	Mid-grade bag
A2	Loose	3	600–800	Standard leaf blend
A6	Loose	3	600–800	Standard loose blend
A8	Bag	3	600–800	Standard blend
A13	Loose	3	500–700	Processing-derived aroma
A16	Cup bag	3	600–800	Fast infusion

**Table 3:** Hyperparameter search grids for the six classical classifiers.

Model	Search Space
SVM	C∈{0.1,1,10,100}; kernel ∈ {linear, RBF}; γ∈ {scale, 0.01, 0.1, 1}
k-NN	k∈{3,5,7,9,11}; weights ∈ {uniform, distance}; metric ∈ {Euclidean, Manhattan}
Rand. Forest	trees ∈{50,100,200}; depth ∈ {None, 5 , 10, 15}; min split ∈{2,5}
XGBoost	trees ∈{50,100,200}; depth ∈{3,5,7}; lr ∈{0.01,0.1,0.3}; subsample ∈{0.8,1}
LightGBM	trees ∈{50,100,200}; depth ∈{3,5,7,−1}; lr ∈{0.01,0.1,0.3}; leaves ∈{15,31,63}
MLP	layers ∈ {(50),(100),(50,25),(100,50),(100,50,25)}; act. ∈ {ReLU,tanh}; $\alpha \in \{10^{-4}–10^{-2}\}$

**Table 4:** Ten-fold stratified cross-validation results for six classical models on PCA-reduced features (*n* = 90).

Model	Accuracy	F1-Macro	F1 High	F1 Medium	F1 Low	Consistency
SVM (OvO)	0.611 ± 0.181	0.610	0.627 ± 0.235	0.576 ± 0.195	0.596 ± 0.284	68.2%
k-NN	0.533 ± 0.156	0.534	0.520 ± 0.237	0.504 ± 0.157	0.525 ± 0.304	68.6%
Random Forest	0.611 ± 0.234	0.604	0.668 ± 0.257	0.466 ± 0.342	0.646 ±0.213	71.0%
XGBoost	0.611 ± 0.174	0.604	0.638 ± 0.204	0.480 ± 0.231	0.668 ± 0.163	75.0%
LightGBM	0.511 ± 0.194	0.505	0.487 ± 0.228	0.426 ± 0.275	0.587 ± 0.173	65.5%
MLP	0.622 ± 0.133	0.624	0.728 ± 0.181	0.523 ± 0.142	0.555 ± 0.303	71.0%

**Table 5:** Per-product consistency for MLP under 10-fold cross-validation. Boldface values in the dominant class column indicate incorrect assignments.

Tea	True	Runs	Dominant	Consistency	Distribution
A3	1	11	1	72.7%	C1:8, C2:2, C3:1
A7	1	11	1	90.9%	C1:10, C2:1
A9	1	2	1	50.0%	C1:1, C2:1
A10	1	3	**2**	66.7%	C2:2, C3:1
A11	1	3	1	66.7%	C1:2, C3:1
A1	2	6	2	50.0%	C1:2, C2:3, C3:1
A4	2	3	**3**	66.7%	C1:1, C3:2
A5	2	5	2	100%	C2:5
A12	2	4	**1**	50.0%	C1:2, C2:2
A14	2	5	2	80.0%	C2:4, C3:1
A15	2	6	**3**	66.7%	C2:2, C3:4
A2	3	7	3	85.7%	C2:1, C3:6
A6	3	5	3	80.0%	C2:1, C3:4
A8	3	6	3	66.7%	C2:2, C3:4
A13	3	5	**2**	80.0%	C1:1, C2:4
A16	3	8	3	62.5%	C1:1, C2:2, C3:5

**Table 6:** Per-product consistency for MS-CNN-Attention under StratifiedGroupKFold 10-fold cross-validation. A check mark (✓) marks products whose dominant (majority-vote) class matches the true grade; a cross (×) marks an incorrect assignment.

Tea	True	Runs	Dominant	Consistency	Distribution	Correct
A3	1	11	1	72.7%	C1:8, C3:3	✓
A7	1	11	1	100%	C1:11	✓
A9	1	2	1	50.0%	C1:1, C2:1	✓
A10	1	3	1	66.7%	C1:2, C2:1	✓
A11	1	3	1	100%	C1:3	✓
A1	2	6	2	100%	C2:6	✓
A4	2	3	2	66.7%	C1:1, C2:2	✓
A5	2	5	2	100%	C2:5	✓
A12	2	4	1	75.0%	C1:3, C2:1	×
A14	2	5	2	100%	C2:5	✓
A15	2	6	2	66.7%	C2:4, C3:2	✓
A2	3	7	3	85.7%	C2:1, C3:6	✓
A6	3	5	3	80.0%	C2:1, C3:4	✓
A8	3	6	3	83.3%	C2:1, C3:5	✓
A13	3	5	1	40.0%	C1:2, C2:1, C3:2	×
A16	3	8	3	100%	C3:8	✓

**Table 7:** Comprehensive model comparison. The final row (Δ vs. MLP) reports the change in the MS-CNN-Attention model relative to the best classical baseline (MLP); “pp” denotes percentage points.

Model	Paradigm	F1-Macro	Accuracy	Consistency	Correct Dominant
SVM (OvO)	Feature	0.610	0.611 ± 0.181	68.2%	10/16
k-NN	Feature	0.534	0.533 ± 0.156	68.6%	8/16
Random Forest	Feature	0.604	0.611 ± 0.234	71.0%	10/16
XGBoost	Feature	0.604	0.611 ± 0.174	75.0%	8/16
LightGBM	Feature	0.505	0.511 ± 0.194	65.5%	9/16
MLP	Feature	0.624	0.622 ± 0.133	71.0%	11/16
MS-CNN-Attn	Raw DL	0.811	0.810 ± 0.198	80.4%	14/16
Δ vs. MLP		+30%	+30%	+9 pp	+3

**Table 8:** Representative tea e-nose studies compared with this work. “Array” denotes a multi-sensor gas array.

Study	Sensing Platform	Gas Sensors	Modeling	Product-Level Consistency
Dutta et al. (2003) [12]	MOS e-nose	Array	ANN on features	No
Yu and Wang (2007) [19]	MOS e-nose	Array	LDA/PCA on features	No
Hidayat et al. (2019) [20]	MOS e-nose	Array	PCA/LDA/SVM on features	No
Wang et al. (2022) [21]	MOS e-nose	Array	Extreme learning machine	No
Hu et al. (2024) [22]	e-nose + Vis–NIR	Array	Chemometric fusion	No
Shi et al. (2025) [23]	e-nose + hyperspectral	Array	Deep feature fusion (GLFNet)	No
Ferreira et al. (2026) [26]	Low-cost e-nose + NIR	Array	PLSDA/LDA/PCA on features	No
Shi et al. (2026) [29]	MOS e-nose	Array	Cross time–frequency deep net	No
**This work**	**Single BME688 MOS**	**Single**	**Feature ML vs. raw-waveform DL**	**Yes**

## Data Availability

The raw data supporting the conclusions of this article will be made available by the authors on request.

## Abbreviations

The following abbreviations are used in this manuscript:

BME688	Bosch Multi-parameter Environmental Sensor
BSEC	Bosch Sensortec Environmental Cluster
CNN	Convolutional Neural Network
GC-MS	Gas Chromatography–Mass Spectrometry
HPLC	High-Performance Liquid Chromatography
IAQ	Indoor Air Quality
k-NN	k-Nearest Neighbors
MLP	Multi-Layer Perceptron
MOS	Metal–Oxide–Semiconductor
MS-CNN	Multi-Scale Convolutional Neural Network
OAV	Odour Activity Value
ORAC	Oxygen Radical Absorbance Capacity
PCA	Principal Component Analysis
RMS	Root Mean Square
SE	Squeeze–Excitation
SVM	Support Vector Machine
VOC	Volatile Organic Compound
